# Immune–fibroblast ligand–receptor axes in post-infarction cardiac remodeling: convergent and divergent mechanisms compared with cancer-associated fibroblasts

**DOI:** 10.3389/fimmu.2026.1992368

**Published:** 2026-10-28

**Authors:** Hong Lu, Xiao-long Tao, Ding-ming Song, Yi-bo He, Lan-zhi Zheng

**Affiliations:** 1 The First Affiliated Hospital of Zhejiang Chinese Medical University (Zhejiang Provincial Hospital of Chinese Medicine), Hangzhou, China; 2 Department of Gastroenterology and Hepatology, The First Affiliated Hospital of Zhengzhou University, Zhengzhou, China; 3 Department of Urology, The Second Affiliated Hospital of Zhejiang University, Hangzhou, China

**Keywords:** cancer-associated fibroblast, cardiac fibroblast, ligand–receptor, macrophage, myocardial infarction

## Abstract

Myocardial infarction-triggered cardiac fibrosis is a cardinal feature of maladaptive ventricular remodeling and a principal contributor to heart failure pathogenesis. Enabled by single-cell RNA sequencing and spatial transcriptomics, accumulating evidence demonstrates that post-infarction fibrosis represents an actively coordinated biological program rather than a passive wound-healing event, which is governed by ligand-receptor-mediated crosstalk between immune cells and cardiac fibroblasts. Pioneering work has established monocyte-derived macrophages as a predominant cellular source of pro-inflammatory interleukin-1β; upon binding its cognate receptor on fibroblasts, this cytokine instigates the generation of pro-fibrotic fibroblast subsets within spatially segregated niches of the infarcted myocardium. Multiple additional ligand-receptor modules, encompassing osteopontin-CD44/integrin, transforming growth factor-β, platelet-derived growth factor, as well as growth arrest-specific protein 6-MER tyrosine kinase, further fine-tune fibroblast activation trajectories in a spatiotemporally-restricted fashion. Intriguingly, a substantial fraction of these ligand-receptor interactions are conserved in cancer-associated fibroblast biology, wherein osteopontin-producing macrophages and activated fibroblasts collaboratively build pro-tumor stromal microenvironments. We consolidate recent multi-omic findings regarding immune-fibroblast ligand-receptor communication during post-infarction cardiac remodeling, highlight mechanistic analogies to cancer-associated fibroblast activation, and discuss therapeutic prospects arising from targeting these conserved signaling nodes across cardiac and neoplastic diseases.

## 1 Introduction

Myocardial infarction remains a major cause of morbidity and mortality worldwide, and survivors remain at risk of heart failure due to progressive cardiac fibrosis (1). Post-infarction remodeling proceeds from an early inflammatory phase, marked by neutrophil and monocyte infiltration, to a reparative phase involving fibroblast activation, myofibroblast differentiation, and extracellular matrix deposition (1, 2). Although this response initially limits ventricular rupture, persistent fibrosis causes diastolic dysfunction, arrhythmia, and progressive heart failure (2, 3). Fate-mapping studies indicate that resident fibroblasts proliferate and adopt specialized scar-forming states, while perivascular progenitors also contribute to injury-induced fibrosis (4, 5).

Recent evidence shows that cardiac fibrosis is not solely a fibroblast-autonomous process but is shaped by paracrine and juxtacrine signals from the immune microenvironment (6). Macrophages regulate fibroblast phenotypes through soluble cytokines, including IL-1β, TNF-α, TGF-β, and IL-6, and through cell-associated and contact-dependent ligand–receptor interactions (6, 7). Single-cell RNA sequencing and spatial transcriptomics have mapped these interactions and identified disease-associated fibroblast states, including FAP^+^/POSTN^+^ and THBS4^+^ populations, within defined anatomical niches (3, 8, 9). Many of these markers and signaling components, including FAP, FSP1, PDGFRα/β, and POSTN, are also characteristic of cancer-associated fibroblasts (CAFs) (10, 11). In tumors, SPP1^+^ macrophages interact with FAP^+^ FSP1^+^ CAFs to form immunosuppressive and pro-tumorigenic niches (12, 13). This raises the question of whether cardiac fibroblasts and CAFs share not only phenotypic markers but also upstream ligand–receptor circuits. Here, we synthesize recent multi-omic evidence on immune–fibroblast communication after myocardial infarction, compare its convergent and divergent features with CAF biology, and discuss translational opportunities for ligand–receptor-targeted antifibrotic therapies.

## 2 Immune–fibroblast ligand–receptor communication in the post-infarction heart

### 2.1 Spatial and temporal maps of immune–fibroblast crosstalk niches

Kuppe et al. (3) generated a spatial multi-omic atlas of the human infarcted myocardium by integrating snRNA-seq, snATAC-seq, and spatial transcriptomics from 23 patients. The study identified distinct niches in the infarct core, border zone, and remote myocardium, showing preferential co-localization of CCR2^+^ monocyte-derived macrophages with FAP^+^/POSTN^+^ fibroblasts in the infarct core, whereas CCR2^−^ resident macrophages were associated with APOE^+^/AGT^+^ fibroblasts in remote regions (3, 14). These distributions reflect functional differences between recruited pro-inflammatory macrophages and resident macrophages that support tissue homeostasis and repair (14). Complementary single-cell atlases have further defined the cellular composition of the adult human heart (15, 16). Immune–fibroblast crosstalk also shifts over time. During the early response to injury, SPP1-high macrophages dominate. Later, reparative subsets expand. These include osteopontin-producing macrophages driven by the IL-10–STAT3–galectin-3 axis (17). They also include partially self-renewing resident-derived populations that limit adverse remodeling (18, 19). Together they shape the repair phase (20, 21). These states are best viewed as overlapping transcriptional programs rather than distinct lineages. So SPP1-high macrophages should not automatically be treated as separate from the reparative pool. Trajectory-based ligand–receptor inference also hints that SPP1-high macrophages may activate fibroblasts through CD44 and integrins (22). But this remains a computational prediction. It has not yet been shown to hold in functional experiments. Complementary single-cell studies have clarified the fibroblast states shaped by these signals. Koenig et al. (8) integrated single-cell and single-nucleus RNA-sequencing data from 45 human hearts and showed that fibroblasts diversify in disease into subpopulations marked by POSTN, THBS4, EDNRA and CCL2 that expand in failing hearts. Amrute et al. (7), multi-omic profiling healthy, acutely infarcted and chronically failing hearts, identified a disease-associated fibroblast trajectory that diverges into myofibroblast-like and pro-fibrotic populations; the latter resemble matrifibrocytes (4) and contribute to the POSTN^+^ rather than the myofibroblast lineage. These findings indicate that immune signals direct fibroblasts toward distinct activation states rather than acting on a uniform cell population. Similar single-cell and spatial studies in renal cell carcinoma and keloid fibrosis support the broader applicability of this analytical framework to immune–stromal organization across diseases (23, 24).

Neutrophils, the first leukocytes recruited to the infarct, represent an additional and still under-mapped node of immune–fibroblast communication. The macrophage-mediated route is supported by depletion studies in mice: neutrophil depletion delayed healing and increased fibrosis by polarizing macrophages toward a reparative, efferocytosis-competent phenotype (25), and NET-mediated macrophage polarization similarly promotes adverse remodeling after MI (26). The direct route is supported independently. In patients with STEMI, NETs measured at the culprit site were associated with fibrocyte responses and adverse remodeling (27), and genetic ablation of PAD4-dependent NET formation protects aged mice from left ventricular interstitial collagen deposition and decline in cardiac function (28). Together, these findings indicate that neutrophils can shape fibroblast activation through both macrophage-dependent and macrophage-independent pathways, although the relative contribution of each route across the phases of infarct healing remains to be defined.

### 2.2 Key ligand–receptor axes driving fibroblast activation

Immune-derived ligands do not induce a single activated fibroblast state. Distinct signals drive distinct phenotypes. TGF-β–Smad2/3 signaling promotes differentiation into ACTA2^+^ myofibroblasts specialized for contractile matrix production, a response confirmed by fibroblast-specific Smad2/3 deletion in mice (1, 29, 30). IL-1β–IL-1R signaling instead drives the emergence of FAP^+^/POSTN^+^ fibroblasts, which lineage tracing identifies as transcriptionally distinct from conventional myofibroblasts (7). Fibroblast responses further include inflammatory activation, proliferation, matrix production and matrix remodeling, and these outcomes should not be treated as interchangeable. Single-cell profiling of diseased human hearts has resolved discrete chemokine-expressing fibroblast states, including a CCL2^+^ population expanded in heart failure (8), single-nucleus RNA sequencing of the infarcted mouse heart has likewise resolved an inflammatory fibroblast cluster whose TAK1 dependence was established in isolated cardiac fibroblasts using bulk RNA sequencing and protein analysis (31). Spatial multi-omic mapping of the infarcted heart organizes these states into defined immune–fibroblast niches (3). The axes discussed below are therefore described together with the specific fibroblast response they are known or proposed to elicit.

#### 2.2.1 The IL-1β–IL-1R axis: a central inflammatory input

The IL-1β–IL-1R axis has emerged as a central ligand–receptor pair driving post-infarction fibroblast activation. Amrute et al. (7) performed multi-omic profiling of human infarcted and chronically failing hearts and demonstrated that CCR2^+^ macrophages secrete IL-1β, which signals through IL-1R on cardiac fibroblasts to drive the emergence of FAP^+^/POSTN^+^ fibroblasts (**Figure 1**). Conditional deletion of IL-1β in CCR2^+^ monocytes/macrophages, conditional deletion of IL-1R in fibroblasts, and anti-IL-1β monoclonal antibody treatment each reduced FAP^+^/POSTN^+^ fibroblast abundance, attenuated fibrosis, and improved cardiac function in mouse models of combined angiotensin II/phenylephrine infusion, establishing a requirement for this axis in that setting (7). Because these perturbations were not performed in infarct models, their extension to infarct healing remains an extrapolation supported so far by human spatial associations (3, 7).

**Figure 1:**
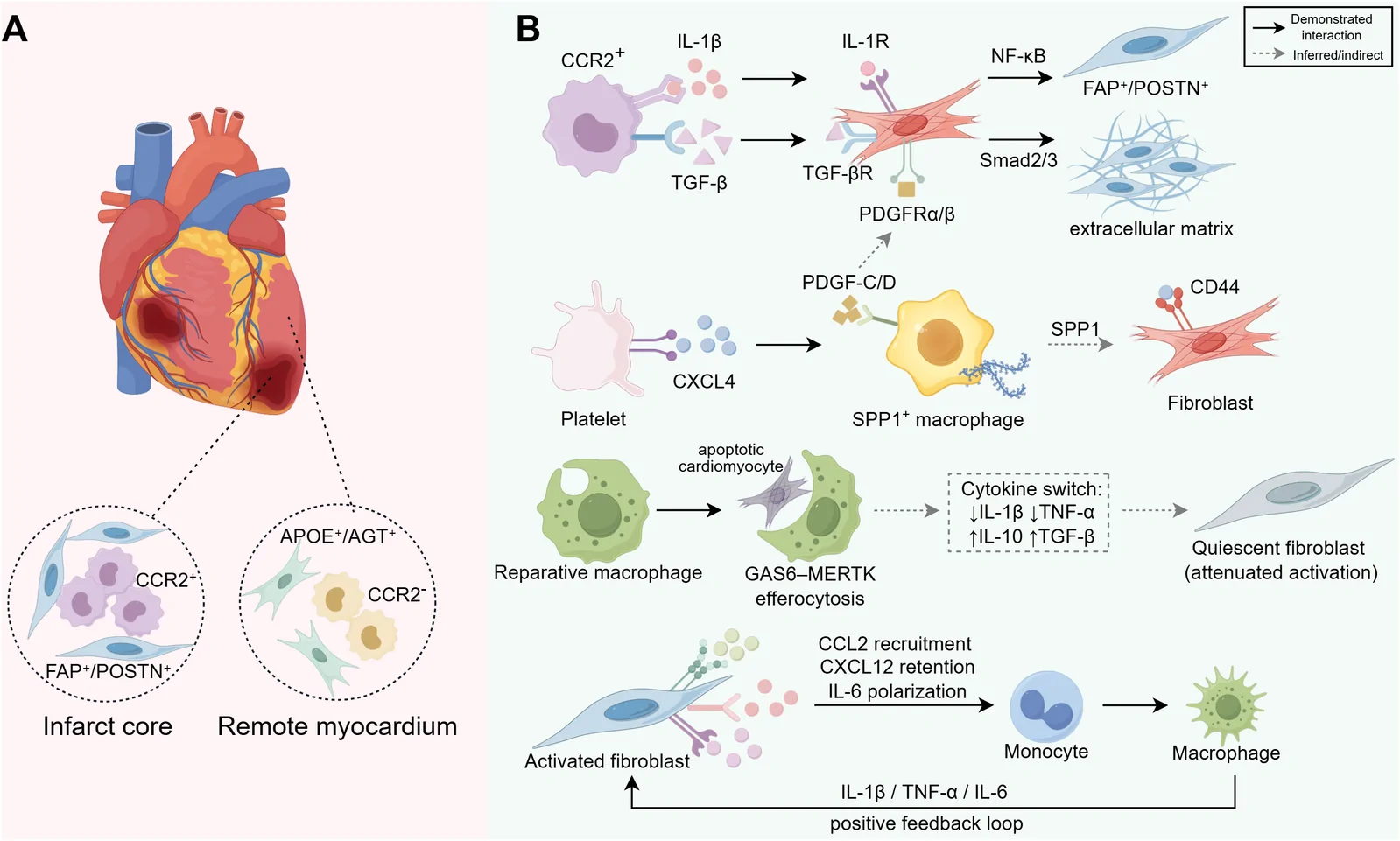
Immune–fibroblast ligand–receptor crosstalk orchestrates cardiac fibroblast activation after myocardial infarction. **(A)** Spatial organization of immune–fibroblast communication niches in the infarcted heart. In the infarct core, CCR2^+^ monocyte-derived macrophages co-localize with FAP^+^/POSTN^+^ activated fibroblasts, whereas in the remote myocardium, CCR2^−^ resident macrophages associate with APOE^+^/AGT^+^ fibroblasts. **(B)** Key ligand–receptor axes and regulatory circuits of fibroblast activation after infarction. CCR2^+^ macrophages secrete IL-1β and TGF-β, which engage IL-1R (activating NF-κB) and TGFβR (activating Smad2/3) on cardiac fibroblasts to drive the FAP^+^/POSTN^+^ pro-fibrotic state and extracellular matrix (ECM) deposition. PDGF-C/D signaling from SPP1^+^ macrophages to fibroblasts (inferred from human spatial multi-omics) engages PDGFRα/β. Platelet-derived CXCL4 instructs the differentiation of SPP1^+^ macrophages, whose secreted SPP1 is predicted to engage CD44 on fibroblasts. Reparative macrophages clear apoptotic cardiomyocytes through GAS6–MERTK-mediated efferocytosis; the associated cytokine switch (reduced IL-1β and TNF-α, increased IL-10 and TGF-β) indirectly attenuates inflammatory fibroblast activation. Conversely, activated fibroblasts release CCL2, CXCL12 and IL-6 to recruit, retain and polarize monocytes that differentiate into macrophages, while macrophage-derived IL-1β, TNF-α and IL-6 feedback on fibroblasts, closing a proposed positive feedback loop; whether this loop operates *in vivo* as a single self-sustaining circuit remains to be tested. Solid arrows denote experimentally supported interactions; dashed arrows denote inferred or indirect effects.

Mechanistically, IL-1β signaling activates NF-κB-dependent pathways in fibroblasts, promoting proliferation and extracellular matrix gene expression. In parallel, TGF-β, also supplied by macrophages in the infarcted heart (1, 3), activates Smad3-dependent pathways in fibroblasts that facilitate migration, myofibroblast transdifferentiation, and collagen synthesis. Macrophage-derived IL-6 acts on neighboring cells in a paracrine manner, and selective blockade of IL-6 trans-signaling attenuated inflammatory infiltration and adverse remodeling after reperfused MI (32), supporting a contribution of IL-6 signaling to the fibrotic network, although its specific action on fibroblast STAT3 *in vivo* remains to be isolated (1, 6). Clinical evidence points in the same direction. In the CANTOS trial, canakinumab reduced recurrent cardiovascular events in atherosclerotic patients with residual inflammatory risk (33). That trial was not designed to measure post-infarction fibrosis or macrophage–fibroblast signaling. Separately, anakinra has been tested in small early-phase trials. The settings included prevention of adverse remodeling after acute MI and recently decompensated heart failure (34, 35). Results were encouraging on inflammatory markers and exploratory imaging endpoints. But there is no definitive evidence that Anakinra regresses fibrosis.

#### 2.2.2 The SPP1–CD44/integrin axis

Osteopontin is a matricellular glycoprotein highly expressed by activated macrophages and implicated in tissue repair and fibrosis across multiple organs. SPP1-high macrophages are a conserved feature of the post-MI microenvironment, enriched early after injury and declining during the fibrotic transition (20, 21). A recent study identified CXCL4, released largely by platelets, as a master instructor of profibrotic SPP1^+^ macrophage differentiation, and showed that loss of CXCL4 ameliorated fibrosis after both heart and kidney injury, highlighting platelets as unexpected upstream regulators of this axis (36). SPP1 signals through multiple receptors, including CD44 and integrins, promoting fibroblast chemotaxis, activation, and extracellular matrix production (22, 37).

Loss-of-function studies illustrate the context-dependent roles of osteopontin across cardiac disease models: osteopontin deficiency attenuates fibrosis and pathological remodeling in angiotensin II-induced cardiac hypertrophy (37), whereas osteopontin-producing reparative macrophages are required for optimal healing after MI (17); therapeutic SPP1 silencing in TREM2^+^ cardiac macrophages similarly suppresses atrial fibrillation in preclinical models (38). Within the post-MI heart, the interpretation of these findings requires care. Shirakawa et al. showed that the IL-10–STAT3–galectin-3 axis drives SPP1 expression in reparative CD206^+^ macrophages, and that SPP1 deficiency did not prevent the emergence of galectin-3^hi^ CD206^+^ macrophages but reduced collagen expression and impaired healing (17). SPP1 therefore marks and supports a reparative macrophage program rather than initiating inflammatory polarization. Whether sustained SPP1 expression causally maintains a profibrotic macrophage state, or merely marks macrophages already committed to that program, remains unresolved (17, 22).

#### 2.2.3 The TGF-β–TGFβR and PDGF–PDGFR axes

TGF-β is the canonical pro-fibrotic cytokine in cardiac remodeling, with roles in the infarcted heart established over decades of mechanistic work (29). Cardiac fibroblasts express TGFβR1/2, and TGF-β, produced in the injured heart by macrophages among other cellular sources, as shown by human spatial multi-omic mapping (1, 3), drives Smad2/3-dependent transdifferentiation into α-SMA^+^ myofibroblasts, collagen synthesis, and extracellular matrix cross-linking (1, 29). The PDGF system provides complementary mitogenic signals. In human infarcts, spatial multi-omic analysis detected increased PDGF-C and PDGF-D signaling from SPP1^+^ macrophages to fibroblasts in ischemic samples (3). In mice, PDGF-B/PDGFRα/β signaling is induced in healing infarcts, where receptor-level blockade reduces collagen deposition but impairs vascular maturation, indicating that mitogenic signals are tightly coupled to vascular repair (39), and genetic reduction of PDGFRα^+^ fibroblasts is tolerated at baseline while attenuating injury-induced matrix deposition (40). Together, these axes illustrate a recurrent logic: a differentiation signal plus a mitogenic signal jointly convert a quiescent fibroblast into an expanding pool of matrix-producing effectors (1). In addition, macrophage-derived CCL24 promotes cardiac fibrosis through fibroblast CCR3, a direct chemokine-receptor axis linking myeloid cells to fibroblast recruitment in pressure-overload heart failure (41).

#### 2.2.4 The GAS6–MERTK axis: resolution-phase control of fibroblast activation

The receptor tyrosine kinase MERTK and its ligand GAS6 play essential roles in macrophage efferocytosis and resolution of inflammation. MERTK acts on macrophages, not on fibroblasts. So its influence on fibroblast activation is indirect. It works through the cytokine switch that comes with efferocytosis. During this switch, IL-1β and TNF-α fall, while IL-10 and TGF-β rise (42, 43). In the post-MI heart, MERTK on reparative macrophages is required for timely clearance of apoptotic cardiomyocytes, which in turn promotes an anti-inflammatory cytokine switch and facilitates fibroblast-mediated tissue repair (42, 44). Loss of MERTK function leads to defective efferocytosis, secondary necrosis, and sustained pro-inflammatory signaling that perpetuates fibroblast activation, and ADAM17-mediated cleavage of MERTK during ischemia–reperfusion similarly compromises repair (43, 44). The efferocytosis axis extends beyond MERTK: macrophage AXL, a related TAM(tumor-associated macrophage)-family receptor tyrosine kinase, promotes inflammation rather than resolution after reperfused infarction (45). Efferocytosis-associated receptors are therefore not uniformly reparative and must be targeted with receptor-level precision.

#### 2.2.5 Bidirectional signaling: fibroblasts as immune regulators

Activated cardiac fibroblasts themselves express immune-regulating ligands with distinct functions. CCL2 recruits CCR2^+^ monocytes, CXCL12 promotes retention of CXCR4^+^ myeloid cells, IL-6 acts on both myeloid cells and fibroblasts, and colony-stimulating factors support macrophage survival and differentiation (1, 46). Fibroblasts activated by pressure overload express CCL2 and recruit inflammatory Ly6C^hi^ monocytes, a function demonstrated by fibroblast-specific deletion of the NF-κB activator IKKβ (47). Consistently, IL-1β blockade reduced CCL2 expression in fibroblasts in the study that established the IL-1β axis (7). In the infarct model, fibroblast-specific deletion of TAK1, which propagates IL-1β and TNF-α signaling, reduces immune cell recruitment after non-reperfused MI in male mice, whereas it did not alter remodeling in pressure overload (31). Through these outputs fibroblasts can reinforce monocyte recruitment and macrophage polarization. Indeed, a complete self-amplifying fibroblast–macrophage signaling circuit has been identified in the infarcted heart. Within this cascade, macrophage-derived IFN-β triggers STAT1 signaling in fibroblasts to drive secretion of CCL2, CCL7 and CCL12; these chemokines recruit additional IFN-β-expressing macrophages (48), a finding also observed during *in vivo* cardiac reprogramming. Whether the IL-1β- and IL-6-centered loop highlighted here operates with the same circularity as a single circuit *in vivo* remains to be tested. Fibroblast-derived matrix remodeling products can also engage Toll-like receptors on macrophages and amplify immune activation (1). This bidirectional architecture has therapeutic implications: interrupting a single direction of the loop may suffice to break the cycle when immune cells dominate early after injury, whereas established fibrosis may require simultaneous targeting of both directions—a concept paralleled by the co-targeting of TAMs and CAFs in oncology (10).

### 2.3 Cardiac fibroblasts versus cancer-associated fibroblasts: convergent mechanisms, divergent outcomes

#### 2.3.1 Shared molecular circuitry

The parallels between cardiac fibrosis and tumor stroma are striking at the molecular level. Both contexts feature SPP1^+^ macrophages as key sources of pro-fibrotic ligands; FAP^+^ FSP1^+^ activated fibroblasts as the effector population; TGF-β and IL-1β as central regulatory cytokines; and spatial co-localization of immune and fibroblast populations within defined niches (3, 10, 12). In colorectal cancer, spatial transcriptomics has revealed that SPP1^+^ macrophages and FAP^+^ FSP1^+^ CAFs form co-dependent niches that promote tumor invasion and immune evasion (12)—a cellular architecture remarkably similar to the CCR2^+^ macrophage–FAP^+^/POSTN^+^ fibroblast niche in the infarcted heart. Single-cell atlases of colorectal cancer and hepatocellular carcinoma, together with single-cell dissection of the cirrhotic liver, confirm that myeloid–stromal cellular niches are a general organizing principle of both injured and neoplastic tissues (13, 49, 50).

CAF heterogeneity itself mirrors the emerging taxonomy of activated cardiac fibroblasts. In pancreatic cancer, inflammatory fibroblasts and myofibroblastic CAFs represent distinct functional states with different spatial distributions and secretomes (51), just as the post-MI heart harbors parallel myofibroblast and matrifibrocyte-like populations (4, 8). Both fields have therefore converged on the same conceptual framework: fibroblast activation is a spectrum of states shaped by the local ligand environment rather than a single binary transition (10, 11). Recent work on CAF plasticity in gastric cancer further shows that CAF states are dynamically remodeled by disease-associated signals and can contribute to therapeutic resistance, reinforcing the view that CAF identity is context-dependent rather than fixed (52). Metabolic programs, particularly lipid metabolism, can further couple CAF states to immune escape in the tumor microenvironment, suggesting that conserved ligand–receptor circuitry may be modulated by local nutrient and metabolic conditions (53). Recent studies further show that FAPα^+^ CAFs are closely integrated with immunoregulatory tumor microenvironments (54), that the spatial positioning and matrix programs of CAFs can promote T-cell exclusion in human tumors (55), and that broader CAF–immune-cell crosstalk contributes to tumor immune regulation and therapeutic resistance (56). Certain aspects of this comparison are context-dependent. In pancreatic cancer, IL-1β-driven JAK/STAT signaling and TGF-β antagonize one another to generate inflammatory versus myofibroblastic CAF states (51, 57), indicating that the same ligands can yield distinct fibroblast programs depending on their balance. Conversely, depletion of CAFs can accelerate tumor progression and reduce survival, demonstrating that stromal populations may also restrain disease (58). A comparable antagonism between IL-1β-driven inflammatory activation and TGF-β-driven myofibroblast differentiation has been described in the injured heart (7, 29), but whether it controls fibroblast fate in the same manner remains unknown. **Table 1** summarizes the principal ligand–receptor axes shared between the two settings.

**Table 1:** Comparison of key immune–fibroblast ligand–receptor axes in post-infarction cardiac fibrosis vs. cancer-associated fibroblast activation.

Axis	Sender → receiver	Cardiac fibrosis (post-MI): findings and experimental context	CAF context	Convergence/divergence	Evidence strength (cardiac)
IL-1β–IL-1R	CCR2^+^ macrophage → fibroblast	IL-1β required for expansion of FAP^+^/POSTN^+^ fibroblasts; conditional ligand and receptor deletion plus anti-IL-1β antibody in angiotensin II/phenylephrine-infusion mice; human spatial co-localization in infarct core (3, 7)	TAM-derived IL-1β drives an inflammatory CAF program; antagonized by TGF-β-driven myofibroblastic differentiation (51, 57)	Partially convergent: myeloid→fibroblast direction conserved; downstream fibroblast programs context-dependent (51, 57)	Strong *in vivo* perturbation, but in pressure-overload rather than infarct models; human evidence observational (3, 7)
SPP1–CD44/integrin	SPP1-high macrophage → cardiac fibroblast	SPP1–CD44/integrin signaling inferred from trajectory and spatial analyses; osteopontin-producing reparative macrophages (IL-10–STAT3 axis) required for optimal repair (17, 20–22)	SPP1^+^ TAMs → FAP^+^ FSP1^+^ CAFs; immunosuppressive stromal niches in colorectal and other solid tumors (12, 13)	Similar cellular pairing; functional outcomes context-dependent (repair-supportive vs niche-forming)	Moderate: computational inference and spatial association; no direct ligand-receptor perturbation in infarct models (17, 20, 22)
TGF-β–TGFβR	Macrophage and other sources → fibroblast	Smad2/3-dependent myofibroblast transdifferentiation, collagen synthesis and matrix cross-linking; established over decades of mechanistic work (1, 29)	TAM- and tumor-cell-derived TGF-β → CAF activation, ECM stiffening and immune exclusion (10, 55)	Convergent mechanism, divergent outcome: inflammatory drive resolves and scar persists in the heart, whereas CAF-driven stroma sustains tumor progression (4)	Strong: genetic and pharmacologic evidence across models (1, 29)
PDGF–PDGFRα/β	Induced in healing infarcts → PDGFRα^+^/β^+^ fibroblast and perivascular cells	Human infarcts: increased PDGF-C/D signaling from SPP1^+^ macrophages to fibroblasts (3); murine healing infarcts: PDGF-B/PDGFRα/β induced, receptor blockade reduces collagen but impairs vascular maturation (39)	Tumor-derived PDGF → stromal fibroblast recruitment and perivascular niche formation (11)	Partially convergent: mitogenic and perivascular signals conserved; cardiac role constrained by vascular maturation requirements (39)	Moderate-strong: human spatial inference plus murine receptor-level blockade (3, 39)
GAS6–MERTK	Macrophage → macrophage (autocrine); indirect effect on fibroblasts	Efferocytosis-dependent resolution; MERTK acts on macrophages, curbing inflammatory fibroblast activation via the efferocytosis cytokine switch; ADAM17 cleavage compromises repair (42–44)	MERTK on TAMs mediates efferocytosis of apoptotic tumor cells and may promote immune tolerance (68)	Divergent: resolution-phase signal in the heart vs potential immune evasion in tumors	Moderate: *in vivo* myeloid MERTK studies; fibroblast coupling indirect (42, 44)

#### 2.3.2 Critical divergences: temporal regulation and outcomes

However, key differences exist. In the infarcted heart, fibroblast activation is adaptive at first. The scar it creates is necessary and stays as a stable structure containing long-lived matrifibrocyte-like cells (4). What normally resolves is the inflammatory drive. This happens through MERTK-mediated efferocytosis, IL-10 production, and macrophage polarization toward a reparative phenotype (1, 17). Together, these processes tone down fibroblast activity without erasing it. Maladaptive fibrosis appears when this attenuation fails in the surviving myocardium. In tumors, the opposite is true. Stromal activation is kept alive by persistent oncogenic and inflammatory signals (10). The TGF-β-driven ECM stiffening that is beneficial in cardiac wound healing becomes pathological in cancer by creating a physical barrier to T-cell infiltration (55), so identical molecular machinery yields opposite outcomes depending on whether the initiating injury is self-limiting or sustained.

Furthermore, the therapeutic implications of targeting shared ligand–receptor axes differ fundamentally between the two contexts. In cardiac fibrosis, anti-IL-1β therapy has shown benefit in preclinical models (7), and canakinumab reduced recurrent cardiovascular events clinically (33), although event reduction should not be equated with antifibrotic efficacy against established myocardial fibrosis. In cancer, the same approach aims to disrupt the TAM–CAF axis and sensitize tumors to immunotherapy (54, 56). The most direct example of cross-pollination concerns fibroblast depletion: FAP-targeted CAR-T cells, originally developed to deplete CAFs and remodel tumor stroma (59), were repurposed for cardiac fibrosis, where FAP-directed engineered T cells reduced fibrosis and improved cardiac function in mouse models of cardiac injury (60); *in vivo*-generated CAR-T cells (61) and CD248-targeted BBIR-T cells that selectively eliminate late-activated cardiac fibroblasts (62) have further extended this approach, highlighting the translational value of the immune–fibroblast ligand–receptor framework.

## 3 Therapeutic implications: targeting immune–fibroblast ligand–receptor axes

### 3.1 Repurposing conserved axis-directed therapies

The convergence of cardiac fibrosis and CAF biology on shared ligand–receptor axes suggests several actionable therapeutic strategies. First, anti-IL-1β agents could be repurposed for post-infarction fibrosis. Canakinumab reduced recurrent cardiovascular events and anakinra has been tested in early-phase studies after acute MI (33–35), but no trial has used fibrosis-specific endpoints, so whether stage-stratified blockade modifies the fibroblast response itself remains untested. The remaining challenges are to define the therapeutic window relative to infarct age, the dose that preserves scar integrity, and fibrosis-specific outcome measures (7). Evidence from pulmonary fibrosis indicates that macrophage-restricted secretin-receptor blockade can attenuate fibrotic remodeling, illustrating the therapeutic value of cell-selective receptor modulation in another organ system (63). Second, fibroblast-depleting cell therapies—FAP-targeted or CD248-targeted CAR-T cells—can be adapted from oncology to cardiology (59–62); recent single-cell mapping identifies CD248 as a marker of late-activated cardiac fibroblasts and an organizer of immune–fibroblast interactions, providing a mechanistic and theranostic entry point for such approaches (64). Third, SPP1-neutralizing antibodies or SPP1-directed RNA silencing warrant evaluation for post-MI fibrosis, supported by preclinical studies in cardiac injury and atrial fibrillation (36, 38). Fourth, MERTK agonists or strategies preventing its ADAM17-mediated cleavage could accelerate the transition from inflammatory to reparative macrophage states (43, 44).

### 3.2 Biomarkers and therapeutic timing

Beyond therapy, the ligand–receptor framework offers a rationale for patient stratification and intervention timing. Circulating components of these axes—such as osteopontin, a validated prognostic biomarker in chronic heart failure (65), and galectin-3 (1, 17)—may serve as surrogates of ongoing immune–fibroblast crosstalk and help identify patients with active pro-fibrotic signaling. Timing is equally decisive: preclinical evidence suggests that IL-1β blockade is most effective during the inflammatory phase, before the FAP^+^/POSTN^+^ fibroblast state becomes entrenched, whereas fibroblast-depleting cell therapies target established fibrosis (7, 62); the optimal clinical timing has not been tested. Importantly, systemic inhibition of shared axes carries predictable on-target risks, as illustrated by dose-limiting toxicities encountered with global TGF-β pathway blockade in oncology trials (10), reinforcing the need for tissue-specific delivery.

## 4 Future perspectives

### 4.1 Temporal switches of resolution versus persistence

The explicit comparison with CAF biology reveals that cardiac fibrosis and tumor stroma share more mechanistic commonalities than previously appreciated, but the critical divergence lies in temporal regulation. The inflammatory stimulus that drives cardiac fibroblast activation is self-limiting; the response normally transitioning from inflammation to repair and resolution, whereas tumor stroma is characterized by persistent, self-reinforcing activation (1, 10). The infarct scar itself persists; what differs is whether the immune–fibroblast signaling that generates matrix remains active. Identifying the molecular switches that determine whether fibroblast activation resolves or persists may enable reprogramming of pathological fibrosis toward a regenerative outcome. Candidate switches include efferocytosis-dependent macrophage reprogramming, the balance between IL-10-driven reparative and IL-1β-driven inflammatory signals, and the availability of resolving ligands such as GAS6 (17, 44). Whether any single switch suffices, or whether coordinated multi-axis intervention is required, remains an open question of direct therapeutic relevance.

### 4.2 Validating computationally inferred axes *in vivo*

Many of the ligand–receptor axes discussed here were first nominated by computational inference tools such as CellChat and NicheNet (66, 67), which predict intercellular communication from single-cell transcriptomes but cannot by themselves establish causality. The most convincing studies have therefore combined spatial co-localization with bidirectional genetic perturbation, conditional deletion of the ligand in immune cells and of the receptor in fibroblasts, as performed for the IL-1β–IL-1R axis, providing gold-standard evidence that a given ligand–receptor pair controls fibroblast phenotype *in vivo* (7). Several axes have not yet reached this standard: the SPP1–CD44/integrin axis rests on computational and spatial evidence, the macrophage contribution to TGF-β production in the infarct lacks cell-type-specific loss-of-function support, and IL-1β perturbation has been performed in pressure-overload rather than infarct models. Future validation efforts should therefore combine refined co-culture and organoid systems with *in vivo* genetic models, including lineage-tracing of fibroblast progenitors (5) and of resident versus recruited macrophage subsets (18, 19).

### 4.3 Outstanding questions

Several critical knowledge gaps persist in current research. First, it remains incompletely defined whether immune-fibroblast ligand-receptor networks uncovered in myocardial infarction are conserved across other non-neoplastic cardiac diseases. Preliminary data from heart failure with preserved ejection fraction nevertheless indicate partial overlap in fibroblast-activation programs among these pathological conditions (9). Second, lineage-tracing experiments covering successive disease stages are needed to disentangle the respective functional contributions of tissue-resident and monocyte-recruited macrophages to distinct ligand-receptor axes within the human heart (18, 19). Third, because cultured human fibroblasts fail to recapitulate disease-associated states (7), human-derived platforms such as immune-fibroblast co-culture assemblies and cardiac organoids are needed before clinical translation. Fourth, sex- and age-dependent modulators governing immune-fibroblast crosstalk have yet to be systematically investigated, even though clinical observations have established sexual dimorphism in patient outcomes following myocardial infarction. Fifth, how hypoxia modifies immune–fibroblast ligand–receptor communication in infarcted and tumorous tissue remains open.

## 5 Conclusion

Immune–fibroblast ligand–receptor crosstalk is a central regulatory principle of post-infarction cardiac remodeling. Single-cell and spatial multi-omic technologies now enable mechanistic mapping of the tissue niches and signaling networks that coordinate immune-driven fibroblast activation. The co-localization of CCR2^+^ macrophages with FAP^+^/POSTN^+^ fibroblasts in the infarct core provides anatomical evidence for niche-specific signaling (3, 7). Rather than occurring autonomously or uniformly, fibroblast activation is shaped by immune-derived ligands acting through the IL-1β–IL-1R, SPP1–CD44, TGF-β–TGFβR, PDGF–PDGFRα/β, and GAS6–MERTK axes, directing fibroblasts toward distinct myofibroblast or matrifibrocyte-like states (4, 7). Fibrosis should therefore be understood and treated as a property of the immune–fibroblast interactome rather than of any single pathway. This framework also reveals important parallels with cancer-associated fibroblast biology, including SPP1^+^ macrophage–FAP^+^ fibroblast niches, shared regulatory cytokines, and therapeutic vulnerabilities. However, the immune stimulus that drives post-infarction fibroblast activation is time-limited and normally attenuates after scar formation, whereas tumor stroma is sustained by persistent, self-amplifying activation. This distinction determines therapeutic timing and safety. Future studies should prioritize genetic validation of inferred ligand–receptor axes, lineage-resolved profiling of immune and fibroblast subsets, and biomarker-stratified trials of anti-cytokine and fibroblast-directed therapies.
